# Novel One-Step Multiplex PCR-Based Method for HLA Typing and Preimplantational Genetic Diagnosis of **β**-Thalassemia

**DOI:** 10.1155/2013/585106

**Published:** 2013-04-04

**Authors:** Raquel M. Fernández, Ana Peciña, Maria Dolores Lozano-Arana, Juan Carlos García-Lozano, Salud Borrego, Guillermo Antiñolo

**Affiliations:** ^1^Department of Genetics, Reproduction and Fetal Medicine, Institute of Biomedicine of Seville (IBIS), University Hospital Virgen del Rocío/CSIC/University of Seville, Avenida Manuel Siurot, s/n, 41013 Seville, Spain; ^2^Centre for Biomedical Network Research on Rare Diseases (CIBERER), 41013 Seville, Spain

## Abstract

Preimplantation genetic diagnosis (PGD) of single gene disorders, combined with HLA matching (PGD-HLA), has emerged as a tool for couples at risk of transmitting a genetic disease to select unaffected embryos of an HLA tissue type compatible with that of an existing affected child. Here, we present a novel one-step multiplex PCR to genotype a spectrum of STRs to simultaneously perform HLA typing and PGD for *β*-thalassemia. This method is being routinely used for PGD-HLA cycles in our department, with a genotyping success rate of 100%. As an example, we present the first successful PGD-HLA typing in Spain, which resulted in the birth of a boy and subsequent successful HSC transplantation to his affected brother, who is doing well 4 years following transplantation. The advantage of our method is that it involves only a round of single PCR for multiple markers amplification (up to 10 markers within the *HLA* and 6 markers at the *β-globin* loci). This strategy has allowed us to considerably reduce the optimization of the PCR method for each specific PGD-HLA family as well as the time to obtain molecular results in each cycle.

## 1. Introduction

The thalassemias are a group of autosomal recessive inherited anaemias caused by mutations in the *α*- and *β*-globin genes leading to decreased production of one of the globin chains of haemoglobin (Hb). Mutations in such globin genes are by far the most common group of monogenic disorders worldwide. Recent surveys suggest that between 300,000 and 400,000 babies are born with a serious hemoglobin disorder each year, and although information about the precise world distribution and frequency of these disorders is still limited, there is no doubt that they are going to pose an increasing burden on global health resources in the future [1]. Specifically, over 200 different mutations of the *β*-globin gene cluster have been described, and they result in either absent or reduced synthesis of the *β*-globin chains leading to *β*-thalassemia [2] (http://globin.bx.psu.edu/hbvar/). *β*-thalassemia (OMIM no. 613985) major patients suffer from severe anaemia with marked ineffective erythropoiesis, erythroid marrow expansion, osteopenia, and bone deformities. The high transfusion requirements result in iron overload, and the patients need chelating therapy. The majority of cases will ultimately develop organ damage, in particular, of the heart and liver, with reduced life quality and expectancy [3]. 

The human leukocyte antigen (HLA) system is the name of the major histocompatibility complex (MHC) in humans. The super locus resides on chromosome 6 and contains a large number of genes that encode cell-surface antigen-presenting proteins that, among several functions, play a major role in the immune system function in humans. This complex consists of three regions that contain genes encoding class I, class II, and class III antigens and represents one of the most polymorphic regions of the human genome. HLAs corresponding to MHC class I present peptides from inside the cell. These peptides are produced from digested proteins that are broken down in the proteasomes. In general, these particular peptides are small polymers, about 9 amino acids in length. Foreign antigens attract killer T-cells (also called CD8 positive- or cytotoxic T-cells) that destroy cells. HLAs corresponding to MHC class II present antigens from outside of the cell to T-lymphocytes. These particular antigens stimulate the multiplication of T-helper cells, which in turn stimulate antibody-producing B-cells to produce antibodies to that specific antigen. Self-antigens are suppressed by suppressor T-cells. Finally, HLAs corresponding to MHC class III encode components of the complement system. Diversity of HLAs in the human population is one aspect of disease defense, and, as a result, the chance of two unrelated individuals with identical HLA molecules on all loci is very low. HLA genes have historically been identified as a result of the ability to successfully transplant organs between HLA-similar individuals. In other words, HLA complex is responsible for rejection following organ/tissue transplantation. To date, the only available definitive cure for *β*-thalassemia major is haematopoietic stem cell transplantation (HSCT) from an HLA-identical donor. Matched sibling HSCT for patients under the age of 17 years without major organ damage has been regarded as the best therapy option with a 90% of success, because treatment-related mortality (TRM) is low, and serious morbidity is unusual. Results from matched unrelated and mismatched HSCT are less successful because of a high risk of graft rejection and TRM [4].

Preimplantation genetic diagnosis (PGD) of single gene disorders, combined with HLA matching (PGD-HLA), represents one of the most relevant challenges in reproductive medicine. This strategy has emerged as a tool for couples at risk of transmitting a genetic disease to select unaffected embryos of an HLA tissue type compatible with that of an existing affected child. At delivery, HSC from the newborn umbilical cord blood can be used to treat the affected sibling. This approach is particularly valuable for *β*-thalassemia and other similar life-threatening disorders that require an HLA-compatible HSC donor, where HLA identity seems to provide the best chance of avoiding graft rejection and other serious complications of bone marrow transplantation. To date, very few cases of successful pregnancies and births of healthy HLA compatible donors for patients have been reported [5–10]. Here, we present a new one-step multiplex PCR to simultaneously perform HLA typing and PGD for *β*-thalassemia, which provides important advantages with respect to other methods. To date, we have successfully applied it for 4 couples with children affected by *β*-thalassemia, accounting for a total of 16 PGD-HLA cycles. As an example, we also present the results of the first successful PGD with HLA typing performed in Spain after the application of such method. Subsequent HSCT was performed successfully, and the transplanted sibling is currently doing well, 4 years following transplantation. All the procedures, included HSCs umbilical cord transplantation, were performed at the University Hospital Virgen del Rocio in Sevilla.

## 2. Materials and Methods

### 2.1. Selection of *β*-Globin and HLA Markers

**Figure 1:**
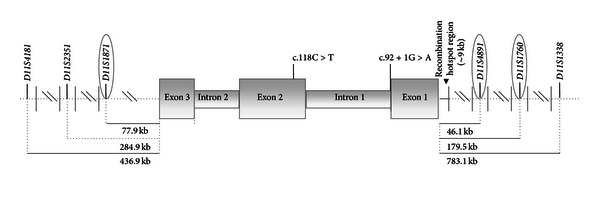
Map of human *β*-globin gene, showing the location of the polymorphic markers that can be tested with our method. The informative STRs for the family here reported are represented as included within an ellipse. The mutations carried by the child and responsible for his clinical picture of *β*-thalassemia are also shown.

A panel of six polymorphic short tandem repeats (STRs) located in the neighboring regions to the *β*-*globin* gene was selected (Figure 1). The selection was based on the heterozygosity values (>30%) detected for each marker in a group of 30 normal controls and in their specific location with respect to the *β*-*globin* gene (3 of them in the 5′region and the other 3 in the 3′region surrounding the gene), warranting the possibility to detect any recombination event at the known recombination hotspot in the beta-globin cluster [11].

**Figure 2:**
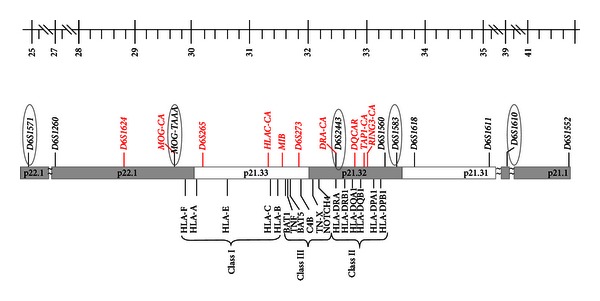
STR markers at the HLA locus that can be simultaneously tested with our method. In red, the new STRs included after the update of the method are indicated following ESHRE recommendations. The informative STRs for the family here reported are represented as included within an ellipse.

**Figure 3:**
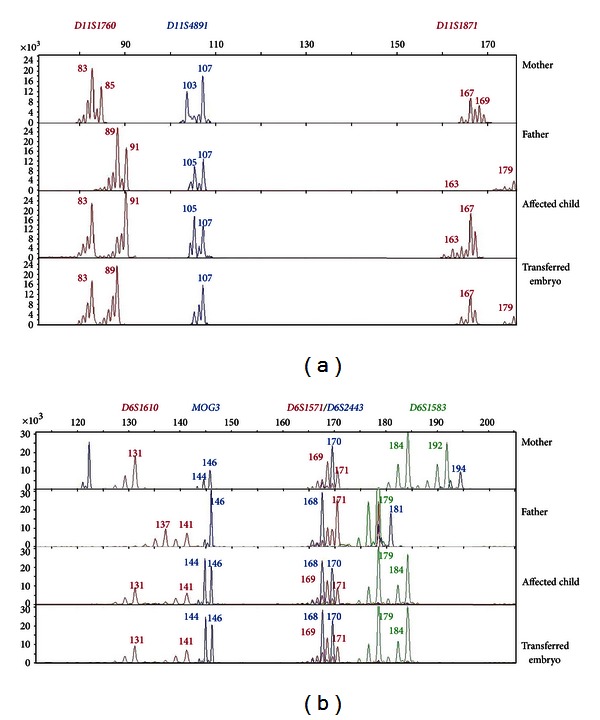
Profiles for the selected informative markers employed in the PGD for *β*-thalassemia (a) with HLA typing (b) in the family here reported.

Regarding HLA typing, a first selection of up to 10 STRs was initially made according to their localization along the HLA locus (Figure 2). The policy was to select, for the subsequent PGD, the maximum number of informative STR markers evenly spaced throughout the HLA complex to obtain an accurate haplotyping, allowing identification of double recombination events, which if not detected may lead to misdiagnosis in HLA typing. Using this panel, we achieved the first successful PGD with HLA typing performed in Spain, which we will also present in this paper. Subsequently and following the ESHRE PGD guidelines [12], the method has been updated with the inclusion of a selection of another 10 markers along the HLA locus (Figure 3).

Primers for the amplification of those markers were designed in order to let all of them be amplified with the same annealing temperature and conditions, using the Primer3 software (http://primer3.sourceforge.net/) (Table 1). As a previous requisite for the final inclusion of the couples in our PGD-HLA program, informativity testing for segregation analyses is always developed on the DNA samples from the corresponding family members (father, mother, and affected child) using standard PCR protocols to identify the “disease haplotypes” and the specific HLA combinations carried by the affected children in the context of their corresponding families.

### 2.2. Multiplex PCR Protocol

A one-step multiplex single-cell fluorescent PCR is used for the simultaneous amplification of several combinations of markers at the HLA and *β*-globin loci, using the QIAGEN multiplex PCR kit (QIAGEN, GmbH; Hilden, Germany) and an adaptation from a protocol previously described [13]. Optimal cell lysis protocol and PCR conditions, further described, were set up on single cells biopsied from supernumerary IVF embryos not suitable for transfer or cryopreservation. Although such cells might give falsely high rates of PCR failure and/or ADO events, achievement of specific optimal PCR conditions under these “not-optimal” circumstances warranted to have a protocol suitable for single cells in real PGD cycles (with a much better quality). The reaction mix contains 0,2 *μ*M each primer, 5× Sol Q, and 2× QIAGEN multiplex PCR master mix, for a final volume of 25 *μ*L. The PCR program is as follows: 15 minutes at 95°C, 10 cycles of 30 seconds at 96°C, 30 seconds at 55°C, 30 seconds at 72°C, followed by 30 cycles of 30 seconds at 94°C, 30 seconds at 55°C, 30 seconds at 72°C, and a final extension of 15 minutes at 60°C. PCR products are analyzed on an ABI3730 automated sequencer (Applied Biosystems, Foster City, CA). 

### 2.3. Assisted Reproductive Techniques and Embryo Biopsy

Controlled ovarian stimulation is performed through a long protocol as previously described [14]. Oocytes are carefully denuded from cumulus cells, and intracytoplasmic sperm injection (ICSI) is used to prevent contamination with residual sperm adhered to the zona pellucida [15, 16]. Blastomere biopsy is performed the morning of day three after fertilization. Laser technology (Octax Laser) is used to create an opening in the zona pellucida, and one blastomere is gently aspirated for each embryo. Cells are transferred into thin walled 0.2 mL PCR tubes containing 2.5 *μ*L of proteinase K/SDS lysis buffer and frozen at −80°C before cell lysis [13]. 

### 2.4. *β*-Globin and HLA Haplotyping of the Embryos

After 30 min at −80°C, cells are lysed by incubation for 90 min at 37°C and 15 min at 65°C. 

HLA typing as well as *β*-globin genetic analysis of the embryos are subsequently performed using the previously selected combination of markers and the described one-step multiplex fluorescent PCR protocol at the single-cell level.

Prior to the analysis, it is established that embryos showing monosomy, trisomy, or uniparental disomy of chromosomes, 6 or 11 will be considered to be abnormal. The embryos with a recombination pattern at the HLA locus are considered to be HLA nonidentical and, therefore, not suitable for transfer.

### 2.5. Case Description of the First Successful PGD with HLA Typing Performed in Spain

A couple of a 29-year-old female and a 35-year-old male, with a 4-year-old son, affected with major *β*-thalassemia, were referred to our department for PGD in combination with HLA typing. Examination of the patient revealed that clinical status of the disease allowed HSCT. After extensive genetic counselling including information about the PGD procedures, success rate and possibility of misdiagnosis inherent to technique, the couple decided to go on with the treatment and was included in our PGD Program. Informed consent concerning PGD and related procedures as well as the fate of the nontransferred embryos was given. 

DNA samples from the affected child and from his parents were obtained with standard protocols and used to perform molecular analyses on both the *β*-*globin* and *HLA loci*.

The disease in this child was found to be due to two different mutations: the IVS-1-nt1[G > A] (HBB:c.92 + 1G > A) from paternal origin, and the CD39[C > T] (HBB:c.118C > T) inherited from his mother (HbVar ID817 and HbVar ID845, resp., according to the human hemoglobin variants and thalassemias database, http://globin.bx.psu.edu/hbvar/) (Figure 1). 

## 3. Results

### 3.1. Analysis of the Efficiency of the Multiplex Protocol

After optimization of the corresponding independent PCR procedures for each marker, we proceeded to perform various assays combining different selections of STRs at both the *β*-*globin* and the *HLA* loci, to analyze the efficiency of the multiplex protocol. Initially, these multiplex protocols were tested using different serial dilutions of genomic DNA extracted from peripheral blood (from 100 ng/mL to 5 pg/mL). Finally, once the final multiplex PCR conditions were adjusted, we tested them on single cells consisting in blastomeres biopsied from supernumerary IVF embryos not suitable for transfer or cryopreservation. Our results showed that different combinations were possible, covering markers at the 5′ and 3′ regions of the *β*-*globin* locus as well as in the different regions of the HLA complex (Figure 3). We tested the combination of up to eight different markers (5 at the HLA locus and 3 in the *β*-globin). Since we had no available DNA from the parents of the embryos donated to this purpose, it was not possible to discriminate between ADO effects or homozygosis for some of the markers tested. Although the efficiency of the PCR varied depending on the marker, no PCR failure was detected for any of them, and the corresponding amplification levels with the final optimized conditions were adequate enough to perform the analysis. 

### 3.2. First Successful PGD with HLA Typing Performed in Spain

After analysis of the STR markers for HLA and *β*-globin haplotypes in the context of the family, we selected five markers for HLA typing (*D6S1571, MOG-TAAA, D6S2443, D6S1583*, and *D6S1610*) and another three for the typing of *β*-globin (*D11S1871, D11S4891*, and *D11S1760)*. The selection of such STRs was made according to their amplification efficiency at the single-cell level, the informativity in the family, and their localization along the *HLA* and *β*-*globin* loci, respectively (see Supplemental Figure available online at http://dx.doi.org/10.1155/2013/585106). Primers sequences for all the markers susceptible to be analysed with our method are provided in Table 1.

The results of the clinical PGD-HLA cycles for this couple are shown in Table 2. All the embryos generated in the PGD-HLA cycles could be successfully diagnosed and HLA-typed. More specifically, two PGD cycles were performed, resulting in a successful pregnancy, with the birth at term of a healthy boy (Figure 2). Cord blood hematopoietic stem cells were obtained and frozen for later use. The stem cells number in the cord blood was high and HSCT was performed three months later, when the child was 7 years old. The child is currently doing well and off all treatments 4 years following transplantation. 

No allele dropout (ADO) and/or contaminations were detected. Recombination events were detected in the paternal *β*-*globin* allele in 1 out of the 28 tested embryos, between markers *D11S1871* and *D11S4891*. Regarding HLA typing, a total of 2 recombinant HLA alleles were identified of either maternal or paternal origin, both of them between markers *D6S1583* and *D6S1610*. 

The remaining unaffected embryos resulting from both cycles that did not achieve enough quality to be cryopreserved, as well as the affected embryos, were retested for both *β*-globin and HLA loci, and the initial results were confirmed in all of them. A total of 12 unaffected and non-HLA-identical embryos suitable to be cryopreserved were vitrified using the VitKit Freeze kit (Irvine Scientific) and the protocol provided by manufacturers.

### 3.3. Update of the Multiplex Genotyping Method

**Figure 4:**
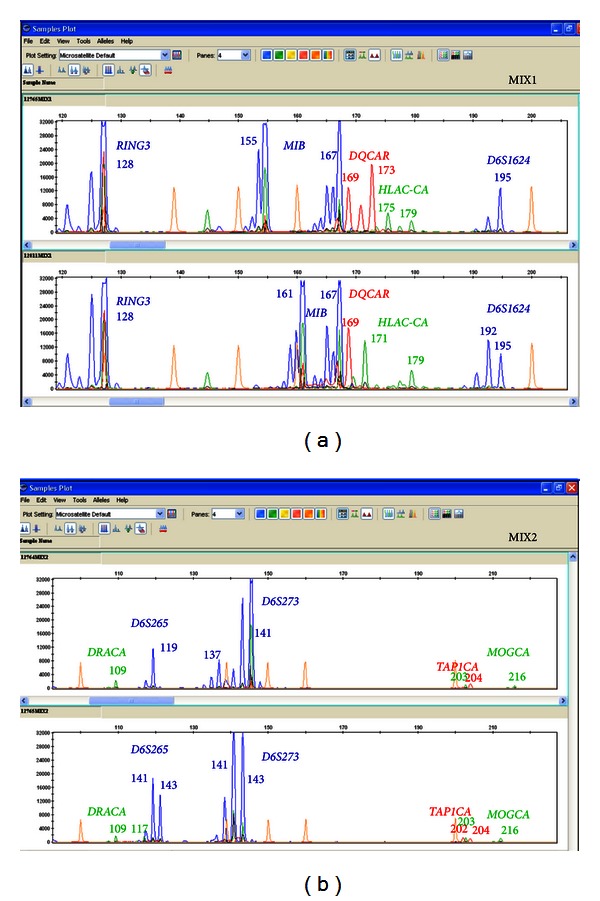
Profiles of 2 different combinations of markers throughout the HLA region after the update of the method on single blastomeres biopsied from nonrelated embryos. Although the efficiency of the PCR varies depending on the marker, the amplification levels obtained for them are adequate to perform the analysis simultaneously. Appropriate zoom of the *y*-axis of the fluorescence level (proportional to the amplification level), let us to observe and analyze all the peaks, corresponding to the different markers.

In 2011, Harton et al. published the ESHRE PGD guidelines in which the general recommendations for HLA typing included a minimum one marker located upstream of HLA-A, minimum one marker between HLA-A and HLA-B, minimum one marker between HLA-B and HLA-DRA, one marker between HLA-DRA and HLA-DQB1, and minimum one marker downstream of HLA-DQB1 [12]. As our initial method did not cover the HLA-HLA-B areas and the HLA-B-HLA-DRA areas, these recommendations prompted us to update our protocol with the inclusion of 10 new markers. We proceeded to perform the same kind of assays previously described for the simultaneous amplification of different combinations of markers, taking into account the policy of covering all the HLA regions recommended by the ESHRE.  Figure 4 shows as an example on the analysis of 2 different combinations of the new HLA markers after performance of the multiplex protocol on single blastomeres. Currently, up to 6 different combinations, including 5 HLA and 2 *β*-globin markers, have been tested and successfully amplified on single blastomeres (Table 3). The goal was to follow the ESHRE PGD guidelines covering all the 5 HLA regions recommended, together with at least 2 markers linked to the *β*-globin locus (5′ and 3′). Of note, we have applied 3 of those combinations (mixes 1 and 2 from Table 3) for the PGD-HLA typing of 3 other couples (14 cycles, 84 embryos), and all the embryos were successfully typed and diagnosed. Again, neither ADO events nor PCR failures were detected, although the efficiency of amplification varied among the markers.

## 4. Discussion

Preimplantation genetic diagnosis in combination with human leukocyte antigen typing offer to families not only the possibility of having unaffected children, but also a new therapeutic option for an affected sibling who has HSCT as the unique option of curative treatment. HLA typing on one cell is complex because the HLA locus is highly polymorphic and large (4 Mb) and recombination within the locus has been observed [17, 18]. Worldwide, HLA testing on preimplantation embryos is routinely performed using STRs. Multiple STRs throughout the HLA region allow 100% accuracy HLA typing and detect possible recombination events, as well as the copy number of chromosome 6 [19]. Efforts should be devoted to the development of a flexible and reliable methodology for PGD/HLA molecular analysis that would shorten as possible the preclinical development time necessary for future cases. For practical and cost-effective reasons, as well as for clinical and psychological ones, it is important that the time to develop a family-specific PCR protocol should be as short as possible. It is critical to design compatible multiple primer sequences and conditions when several PCR reactions may interfere reciprocally in a tube. In this sense, we have optimized a single-cell multiplex PCR method to simultaneously amplify a wide spectrum of STRs within the *HLA* and *β*-*globin* loci, which minimizes the preclinical development time for the families. Usually, multiplex PCR consists of two rounds of amplification. In the first amplification round, all the primers are added together in the reaction mix; in the second amplification round, the first mix is split into distinct mixtures containing two or three primer combinations. Alternatively, multiple displacement amplification (MDA) has been used for whole genome amplification as a previous step to genetic diagnosis and HLA typing of the embryos [8]. The advantage of our approach is that it involves only a round of single PCR for multiple markers amplification, with a considerable reduction of contamination possibility (more probable in two-rounds methods), and workup time to finally obtain a proper typing and diagnosis. Moreover, no ADO events are detected in comparison with other methods reported elsewhere. In addition, and very importantly, this method has demonstrated to be highly versatile, making the incorporation of new markers at both loci feasible. This has let us to include additional markers for subsequent PGD-HLA cycles, taking into consideration the recommendations provided in the ESHRE PGD consortium best practice guidelines [12], which were published after the initial optimization of our method, and even after its application to several PGD cycles in our unit which led to the first successful PGD with HLA typing performed in Spain.

## 5. Conclusions

This strategy has allowed us to considerably reduce the optimization of the PCR method for each specific PGD-HLA family as well as the time to obtain molecular results in each cycle (approximately 4 hours after embryo biopsy). 

## Supplementary Material

Segregation analysis for the STRs at both the *β*-globin and HLA loci in the family. The selected informative STRs for this family are represented in bold.

## Figures and Tables

**Table 1:** Primers sequences for the *β*-thalassemia and HLA markers included in our method.

Marker	Primer	Dye	Sequence	Size of fragment (bp)
HLA typing				
*D6S1571 *	*D6S1571F *	PET	GGACCTACGCATCTGGTG	160–180
	*D6S1571R *		TGGCTCTAATGGTTACTTTTTACA	
*D6S1260 *	*D6S1260F *	NED	ACTGCTCCTGGGCATGGTTG	130–160
	*D6S1260R *		GTACATGCCTTTGTTAACATC	
*D6S1624 *	*D6S1624F *	6FAM	GGAAGTCTTCAGTGGAGAGAGT	200–220
	*D6S1624R *		ACTCCAGGTGTTTGTGGTTT	
*MOG-CA *	*MGCAF *	VIC	TCACCTCGAGTGAGTCTCTTT	205–235
	*MOGCAR *		ACCATGGGTAACTGAAGCAT	
*MOG-TAAA *	*MOG3F *	6FAM	GAAATGTGAGAATAAAGGAGA	125–150
	*MOG3R *		GATAAAGGGGAACTACTACA	
*D6S265 *	*D6S265F *	6FAM	ACGTTCGTACCCATTAACCT	110–125
	*D6S265R *		CGAGGTAAACAGCAGAAAGA	
*HLAC-CA *	*HLACCAF *	VIC	TCCCTAGTAGCTGGGATTACA	155–175
	*HLACCAR *		CGGCAAGAGACTCTGATGA	
*MIB *	*MIBF *	6FAM	CCACAGTCTCTATCAGTCCAGA	155–185
	*MIBR *		TCAGCCTGCTAGCTTATCCT	
*D6S273 *	*D6S273F *	6FAM	GGCCAAAGTTAAAACCAAAC	135–145
	*D6S273R *		GCAACTTTTCTGTCAATCCA	
*DRA-CA *	*DRACAF *	VIC	GATACTTTCCTAATTCTCCTCCTTC	120–140
	*DRACAR *		ATGGAATCTCATCAAGGTCAG	
*D6S2443 *	*D6S2443F *	6FAM	CCATACCAAAGTAAAACCCAG	150–200
	*D6S2443R *		GAGGATGAAGGGAAATTAGAG	
*DQCAR *	*DQCARF *	PET	CTTGGCCAATCAGAATCTTT	150–175
	*DQCARR *		CTGCATTTCTCTTCCTTATCAC	
*TAP1-CA *	*TAP1CAF *	PET	GGACAATATTTTGCTCCTGA	195–215
	*TAP1CAR *		TCATACATCTGCTTTGATCTCC	
*RING3-CA *	*RING3CAF *	6FAM	GGGCCGCAGTTTAAGTAAC	125–135
	*RING3CAR *		TGTTAGGTCAGAACCACAGAA	
*D6S1560 *	*D6S1560F *	6FAM	CTCCAGTCCCCACTGC	225–250
	*D6S1560R *		CCCAAGGCCACATAGC	
*D6S1583 *	*D6S1583F *	VIC	GCCCCTAACCTGCTTCTACTGA	150–200
	*D6S1583R *		GCAGATGGCCCCACTGAC	
*D6S1618 *	*D6S1618F *	NED	GGCCTGAGCAGTGCAT	130–170
	*D6S1618R *		TGATTCCTAATCTGCGGG	
*D6S1611 *	*D6S1611F *	PET	GAGCAAGACTCCATCTCAAA	225–250
	*D6S1611R *		ACCTAAGTTCTCTGAAGGGC	
*D6S1610 *	*D6S1610F *	PET	CCTGGTGAGATAGATGCTTG	100–140
	*D6S1610R *		ATTTCCAGCAGAGCCTTG	
*D6S1552 *	*D6S1552F *	VIC	AGCCTGAACGACAGAACAAG	160–200
	*D6S1552R *		CTGCTTAACTTAGATCTTTGGTAT	
*β*-thalassemia locus				
*D11S4181 *	*D11S4181F *	6FAM	AAGCTTCCTTCACATTCTTACAG	200–225
	*D11S4181R *		GAACTGAGACCAAGAACATTATTCC	
*D11S2351 *	*D11S2351F *	6FAM	GGGCACCTGTAATCCCA	150–180
	*D11S2351R *		AGGAGTCACTGGATCTACTC	
*D11S1871 *	*D11S1871F *	PET	AAGAAGTTGCCCTGATGTCT	160–200
	*D11S1871R *		TAAAAGGAGCTGAATGCACA	
*D11S4891 *	*D11S4891F *	6FAM	GGAAATGGACCTCTGTCTC	75–100
	*D11S4891R *		CTTTTATTCCAGCCCCAC	
*D11S1760 *	*D11S1760F *	PET	GATCTCAAGTGTTTCCCCAC	75–100
	*D11S1760R *		AAACGATGTCTGTCCACTCA	
*D11S1338 *	*D11S1338F *	6FAM	GACGGTTTAACTGTATATCTAAGAC	250–280
	*D11S1338R *		TAATGCTACTTATTTGGAGTGTG	

**Table 2:** Results of the clinical PGD-HLA cycles.

PGD	Cycle 1	Cycle 2
Number of oocytes retrieved	20	25
MII oocytes	17	22
2-pronuclei zygotes	14	18
Embryos biopsied	11	17

Affected	2	8
Carriers	7	3
Noncarriers	2	6

HLA nonidentical	9	14
HLA-identical	2	3

HLA nonidentical and affected	1	7
HLA nonidentical and carriers	6	1
HLA nonidentical and noncarriers	2	6
HLA-identical and affected	1	1
HLA-identical and carriers	1	2
HLA-identical and noncarriers	0	0

Embryos transferred	1	1
Ongoing pregnancy	No	Yes

**Table 3:** Combinations of markers successfully amplified with our one-step multiplex PCR protocol on a single cell, after the update of the method.

	*β*-*Globin*		*HL* *A*				
	5′Region	3′Region	Upstream HLAA	HLAA-HLAB	HLAB-HLADRA	HLADRA-HLADQB1	Downstream DQB1
1	*D11S1338 *	*D11S2351 *	*D6S1624 *	*HLAC-CA *	*MIB *	*DQCAR *	*RING3-CA *
2	*D11S1338 *	*D11S2351 *	*MOG-CA *	*D6S265 *	*D6S273 *	*DRA-CA *	*TAP1A-CA *
3	*D11S4891 *	*D11S4181 *	*D6S1624 *	*HLAC-CA *	*MIB *	*DRA-CA *	*TAP1A-CA *
4	*D11S1760 *	*D11S4181 *	*MOG-CA *	*D6S265 *	*D6S273 *	*DQCAR *	*RING3-CA *
5	*D11S4891 *	*D11S1871 *	*D6S1571 *	*D6S265 *	*MIB *	*D6S2443 *	*D6S1583 *
6	*D11S1760 *	*D11S1871 *	*MOG-TAAA *	*HLAC-CA *	*D6S273 *	*D6S2443 *	*D6S1610 *
