# Supplementary material for: Novel One-Step Multiplex PCR-Based Method for HLA Typing and Preimplantational Genetic Diagnosis of β-Thalassemia
Source: Biomed Res Int. 2013 Apr 4;2013:585106. doi: 10.1155/2013/585106 (PMC3654635; doi:10.1155/2013/585106)
Supplement: Supplementary file 1 — Segregation analysis for the STRs at both the β-globin and HLA loci in the family. The selected informative STRs for this family are represented in bold. [file 585106.f1.ppt]

## Slide 1
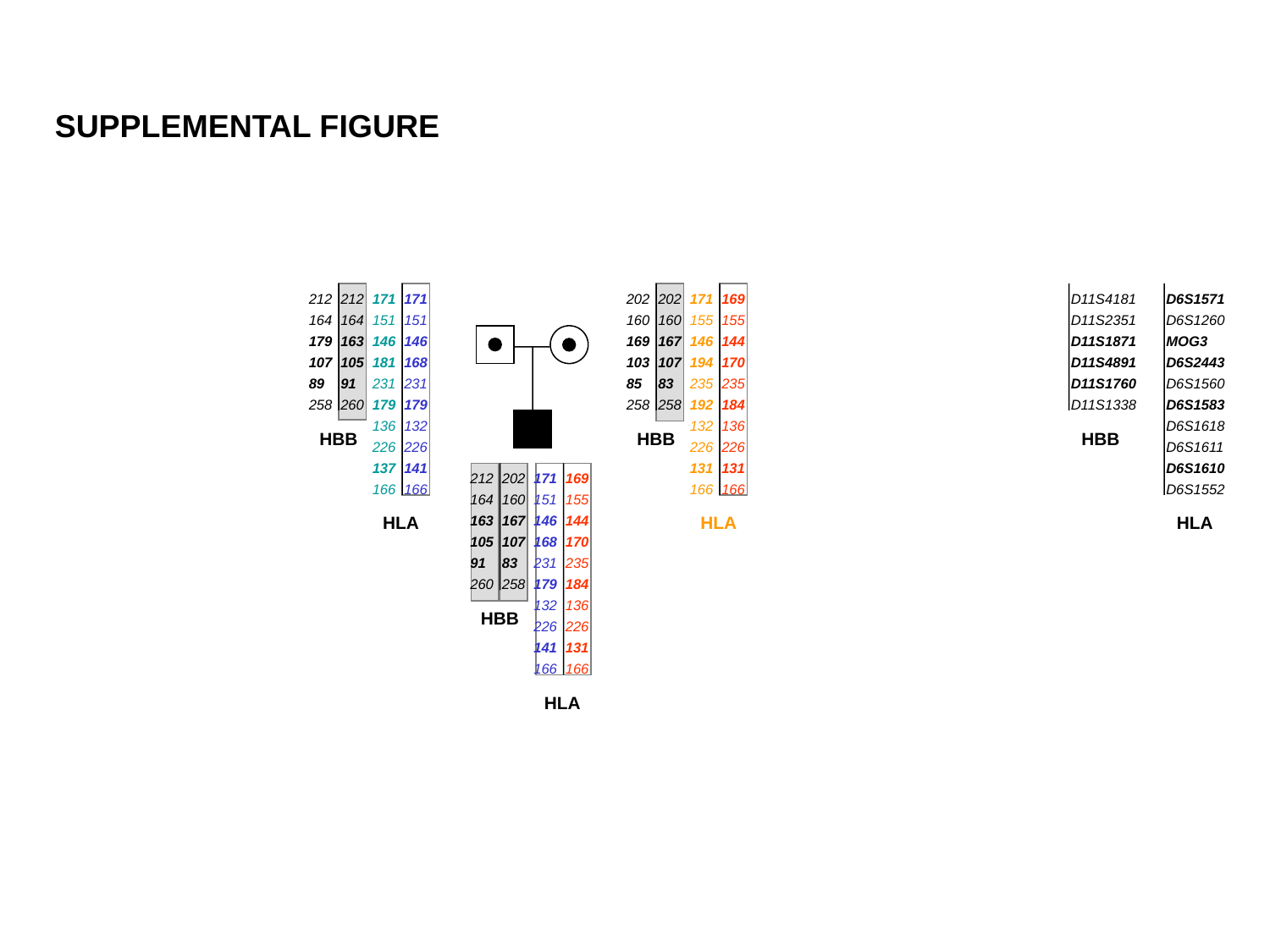

SUPPLEMENTAL FIGURE
212
212
164
163
105
91
260
164
179
107
89
258
HBB
171
151
146
181
231
179
136
226
137
166
171
151
146
168
231
179
132
226
141
166
202
202
160
167
107
83
258
160
169
103
85
258
HBB
171
155
146
194
235
192
132
226
131
166
HLA
169
155
144
170
235
184
136
226
131
166
D11S4181
D11S2351
D11S1871
D11S4891
D11S1760
D11S1338
HBB
D6S1571
D6S1260
MOG3
D6S2443
D6S1560
D6S1583
D6S1618
D6S1611
D6S1610
D6S1552
HLA
212
202
160
167
107
83
258
171
169
155
144
170
235
184
136
226
131
166
164
151
HLA
163
146
105
168
91
231
260
179
132
HBB
226
141
166
HLA
